# Thalassemia in Sub-Saharan Africa: epidemiology, diagnosis, and management – a narrative review

**DOI:** 10.1097/MS9.0000000000003270

**Published:** 2025-04-10

**Authors:** Emmanuel Ifeanyi Obeagu

**Affiliations:** Department of Biomedical and Laboratory Science, Africa University, Mutare, Zimbabwe

**Keywords:** diagnosis, epidemiology, management, Sub-Saharan Africa, thalassemia

## Abstract

Thalassemia is a hereditary blood disorder characterized by the reduced production of hemoglobin, leading to anemia and related complications. In Sub-Saharan Africa, the prevalence of thalassemia, particularly beta-thalassemia, is significant due to genetic predisposition and historical factors such as malaria endemicity. Despite the increasing awareness of the disease, thalassemia continues to be a major public health challenge in the region. Early diagnosis, effective management, and prevention strategies are limited by factors such as poor health care infrastructure, a lack of skilled professionals, and insufficient access to necessary medical treatments. This review article explores the epidemiology, diagnostic approaches, and current management practices for thalassemia in Sub-Saharan Africa. The high prevalence of thalassemia carriers in the region, particularly in malaria-endemic areas, highlights the need for genetic counseling and prenatal screening programs. Furthermore, diagnostic techniques such as hemoglobin electrophoresis and DNA testing are often underutilized due to logistical and financial constraints, leading to late diagnoses and suboptimal care. Treatment options, including blood transfusions and iron chelation therapy, remain inadequate in many parts of Sub-Saharan Africa due to the limited availability of health care resources.

## Introduction

Thalassemia is a group of inherited blood disorders characterized by the abnormal production of hemoglobin, the protein in red blood cells responsible for carrying oxygen throughout the body. The condition results from mutations in the genes responsible for hemoglobin production, leading to anemia, fatigue, and a range of complications related to poor oxygen delivery to tissues. Thalassemia is classified into two main types: alpha-thalassemia and beta-thalassemia. Beta-thalassemia, the more prevalent form in Sub-Saharan Africa, is caused by mutations in the beta-globin gene and can lead to severe forms of anemia, requiring lifelong treatment^[1,2]^. Sub-Saharan Africa has a significant prevalence of thalassemia, with genetic mutations leading to an increased number of carriers in the population. In regions where malaria is endemic, there is a higher frequency of thalassemia, as individuals carrying the trait of thalassemia have some protection against the severe effects of malaria. This genetic relationship between thalassemia and malaria has shaped the genetic landscape of Sub-Saharan Africa over generations, making thalassemia more common in this part of the world compared to other regions. However, despite this high carrier rate, thalassemia remains underdiagnosed, and many affected individuals do not receive proper care or treatment, leading to poor health outcomes^[3,4]^. The burden of thalassemia in Sub-Saharan Africa is compounded by a lack of health care infrastructure and limited access to diagnostic and treatment resources. In many parts of the region, the absence of comprehensive health care systems results in delays in diagnosis, misdiagnosis, and suboptimal management. The disease is often mistaken for other forms of anemia, such as iron deficiency anemia, due to similarities in symptoms. Furthermore, the lack of trained medical personnel and specialized laboratories to perform essential diagnostic tests, such as hemoglobin electrophoresis or DNA testing, hampers early diagnosis and appropriate treatment. This review aims to provide a comprehensive overview of thalassemia in Sub-Saharan Africa, including its epidemiology, diagnostic challenges, management strategies, and future directions for improving care^[5,6]^. The identification and diagnosis of thalassemia are often complicated by the absence of screening programs and public awareness. In many African countries, genetic counseling and prenatal screening for thalassemia are not routinely offered, leading to late diagnosis and the birth of children with severe forms of the disease. When patients present with symptoms, such as fatigue, pallor, and growth retardation, they are often misdiagnosed with other types of anemia, leading to delayed or inappropriate treatment. The lack of awareness among both the general population and health care providers about the genetic nature of thalassemia and its associated risks contributes to the challenges of diagnosis and early intervention^[7,8]^. In Sub-Saharan Africa, beta-thalassemia major, a severe form of the disease, often requires regular blood transfusions to manage the associated anemia. However, the availability of blood transfusion services is limited in many parts of the region due to inadequate blood donation systems, limited access to blood banks, and logistical challenges in transporting blood to rural areas. In addition to transfusions, individuals with thalassemia require iron chelation therapy to prevent iron overload from frequent blood transfusions. These treatments are often expensive and not readily available, making management of the disease difficult for many families, particularly in low-resource settings. As a result, the quality of life for individuals with thalassemia in Sub-Saharan Africa can be severely impacted by the lack of access to adequate care^[9,10]^. Moreover, the only curative treatment for thalassemia, bone marrow or stem cell transplantation, is not widely accessible in Sub-Saharan Africa due to the high cost of the procedure, the lack of transplant centers, and the absence of specialized expertise. While hematopoietic stem cell transplantation (HSCT) can offer a potential cure for beta-thalassemia, the financial and logistical barriers prevent many patients in Sub-Saharan Africa from accessing this treatment option. Consequently, the focus remains on supportive therapies such as blood transfusions and iron chelation, which can improve survival but are not curative^[11]^. To address the growing burden of thalassemia in Sub-Saharan Africa, prevention strategies such as genetic counseling, screening, and public education are vital. Carrier screening, particularly in regions with high malaria prevalence, can help identify individuals at risk of having children with thalassemia. Additionally, prenatal screening for thalassemia can provide couples with information to make informed decisions about family planning. However, the implementation of such programs is hindered by resource constraints, lack of health care personnel, and insufficient awareness among the general public. Efforts to improve the availability of genetic testing and counseling services are necessary to reduce the incidence of severe thalassemia in the region^[12]^. Despite these challenges, some African countries have made progress in addressing the burden of thalassemia. For example, some nations have introduced pilot programs for genetic screening and counseling, which have helped to raise awareness about the disease and identify carriers in high-risk populations. Furthermore, collaboration with international organizations and health care providers can facilitate the sharing of knowledge and resources, leading to the development of more effective prevention and treatment strategies. However, much more needs to be done to ensure that these initiatives reach the broader population and that sustainable solutions are implemented across the continent^[12]^. The role of the government and policymakers is essential in improving thalassemia care in Sub-Saharan Africa. National health policies that prioritize thalassemia as a public health issue can help secure funding for prevention, diagnosis, and treatment programs. Governments should also invest in strengthening health care infrastructure, particularly in rural areas, to improve access to essential diagnostic services, blood transfusions, and iron chelation therapies. Additionally, public health campaigns to raise awareness about thalassemia and its prevention can help reduce the stigma associated with the disease and encourage individuals to seek early diagnosis and treatment^[13]^.

**HIGHLIGHTS**

- Epidemiological trends: Highlights the prevalence and genetic diversity of thalassemia in Sub-Saharan Africa, emphasizing its interplay with malaria-endemic regions and public health challenges.
- Diagnostic challenges: Reviews the limitations in diagnostic infrastructure, including reliance on basic hematological tests, leading to underdiagnosis and mismanagement in resource-limited settings.
- Management strategies: Discusses emerging therapies, limited access to transfusion services, and iron chelation therapy hurdles in the African context.
- Policy gaps: Explores the lack of comprehensive national policies for thalassemia screening and management, stressing the need for targeted interventions.
- Research and advocacy: Advocates for more genetic studies and regional collaborations to improve awareness, early diagnosis, and sustainable management programs.

## Aim

This narrative review aims to explore the epidemiology, diagnostic challenges, and management strategies of thalassemia in Sub-Saharan Africa.

## Justification of the review

Thalassemia represents a significant but often underrecognized public health concern in Sub-Saharan Africa, where genetic predispositions and limited access to health care resources compound the burden of the disease. The review is justified by the need to consolidate existing knowledge and provide a comprehensive assessment of the current state of thalassemia management in the region. While thalassemia is well-documented in other parts of the world, there is a lack of focused and region-specific studies that address the unique challenges faced by Sub-Saharan African countries. This review aims to fill that gap by providing a contextualized understanding of thalassemia in the African setting^[1,2]^. Additionally, Sub-Saharan Africa faces several systemic barriers in health care infrastructure, such as limited access to diagnostic facilities, blood transfusion services, and specialized medical care, which hinder effective thalassemia management. By exploring these challenges and evaluating current practices, this review seeks to highlight critical areas that require urgent attention and improvement. Moreover, the review justifies the need for preventive measures and public health initiatives that can reduce the incidence of thalassemia through early genetic screening and education^[3,4]^. The review also addresses the pressing need for innovations in thalassemia care, such as advancements in genetic screening technologies, bone marrow transplantation, and gene therapy, which have the potential to significantly enhance the management and long-term outcomes for patients in Sub-Saharan Africa. Furthermore, the need for policy reforms, integration of thalassemia management into national health programs, and strengthening of regional collaborations is imperative for ensuring sustainable and equitable access to care. The insights from this review can guide policymakers, health care professionals, and researchers in prioritizing thalassemia as a public.

### Review methods

In conducting this narrative review on thalassemia in Sub-Saharan Africa, a systematic approach was employed to ensure a comprehensive understanding of the topic. The review focused on the epidemiology, diagnosis, and management of thalassemia, synthesizing information from a variety of sources.

### Search strategy

To gather relevant literature, a thorough search was performed using several electronic databases, including PubMed, Google Scholar, Scopus, Web of Science, and African Journals Online (AJOL). The search utilized specific keywords and phrases, such as “Thalassemia AND Sub-Saharan Africa,” “Alpha-thalassemia AND malaria,” and “Diagnosis of thalassemia in resource-limited settings.” This approach aimed to capture a wide array of studies addressing various aspects of thalassemia in the African context.

### Inclusion and exclusion criteria

The review adhered to specific inclusion and exclusion criteria to maintain focus and relevance. Studies published in English from 2000 to 2024 were considered to ensure that the findings were current. This included research articles, review papers, case studies, and clinical guidelines that specifically addressed the epidemiology, genetics, diagnosis, and management of thalassemia in Sub-Saharan Africa. Additionally, studies exploring the relationship between thalassemia and malaria were also included, given the significance of malaria in the region. Conversely, studies that focused exclusively on thalassemia in non-African populations were excluded, unless they provided valuable comparative insights. Furthermore, any articles with limited access to full texts or unverifiable sources, as well as those centered on other hemoglobinopathies without relevance to thalassemia, were not considered.

### Epidemiology of thalassemia in Sub-Saharan Africa

Thalassemia is a hereditary blood disorder with a global distribution, but its prevalence varies across different regions. In Sub-Saharan Africa, thalassemia, particularly beta-thalassemia, is a significant public health issue, primarily due to the high frequency of genetic carriers. This region’s thalassemia prevalence is influenced by historical and evolutionary factors, including the interaction between thalassemia and malaria. The relationship between malaria and thalassemia has led to higher carrier rates in malaria-endemic areas, as individuals who are carriers of thalassemia have a selective advantage against severe forms of malaria. Consequently, Sub-Saharan Africa, which has one of the highest malaria burdens in the world, also shows high levels of thalassemia gene mutations, especially in populations residing in malaria-endemic zones^[8,14]^. The prevalence of thalassemia in Sub-Saharan Africa is particularly notable in countries within the equatorial belt, where malaria transmission is high. Studies estimate that up to 5–10% of people in these areas may carry the thalassemia trait, with varying prevalence rates depending on the specific country or region. For instance, in some countries like Nigeria, Kenya, and Ghana, the carrier rates for beta-thalassemia can range between 5% and 10%. These high carrier rates increase the likelihood of children inheriting the disease, particularly beta-thalassemia major, which can result in severe anemia and require lifelong medical care. Furthermore, the prevalence of thalassemia in Sub-Saharan Africa is often underreported, as the condition is not routinely screened for, and many individuals with mild forms of the disease may remain undiagnosed or misdiagnosed with other types of anemia^[3,15]^. In addition to beta-thalassemia, alpha-thalassemia is also present in Sub-Saharan Africa, although it tends to be less well-studied. Alpha-thalassemia is more common in populations of African descent, particularly in West and Central Africa. The genetic mutations that lead to alpha-thalassemia result in a reduction or absence of the alpha-globin chain, which, like beta-thalassemia, affects hemoglobin production. Although alpha-thalassemia generally causes milder forms of anemia than beta-thalassemia, it can still contribute to health complications, particularly in individuals with severe forms. The coexistence of both alpha- and beta-thalassemia in some populations increases the complexity of thalassemia’s epidemiology in Sub-Saharan Africa, making it essential to improve surveillance and screening programs to better understand the full burden of thalassemia in the region^[16]^. Due to the lack of widespread screening programs and genetic counseling, many individuals with thalassemia in Sub-Saharan Africa are diagnosed late or not at all, particularly in rural and underserved areas. As a result, the full scope of thalassemia’s impact on public health in the region remains unclear. However, the increasing recognition of thalassemia as a significant cause of anemia in affected regions highlights the need for better diagnostic tools and early detection methods. There is a growing awareness among health care professionals and public health authorities about the importance of early genetic screening, particularly in high-risk populations, to reduce the incidence of severe forms of thalassemia and to provide affected individuals with better care and treatment options. With greater access to health care services and improved diagnostic infrastructure, it is possible to more accurately assess the true burden of thalassemia in Sub-Saharan Africa, leading to more effective prevention and management strategies^[17,18]^. Thalassemia’s prevalence in Sub-Saharan Africa is closely linked to malaria-endemic regions, where carrier states offer a protective advantage against severe *Plasmodium falciparum* infections. Alpha-thalassemia is notably widespread, with up to 50% of certain populations carrying related genetic traits. Beta-thalassemia, though less common, has been identified in specific areas. For instance, a study in Kilifi, Kenya, reported a beta-thalassemia carrier frequency of approximately 0.3% within the study population. In Togo, data from 1992 to 1994 indicated a beta-thalassemia prevalence of 597 per 100 000 individuals. These figures highlight the underrecognized presence of thalassemia in the region. The high frequency of alpha-thalassemia variants is primarily attributed to evolutionary pressure from malaria. Co-inheritance with other hemoglobinopathies, such as sickle cell disease, can influence disease severity and complicate clinical management. Population migration and intermarriage further impact the genetic distribution of thalassemia, introducing diverse mutations and combinations. Thalassemia manifests in a spectrum from asymptomatic carriers to severe, transfusion-dependent anemia. Common symptoms include pallor, jaundice, growth retardation, hepatosplenomegaly, and skeletal deformities due to bone marrow expansion. However, these symptoms often overlap with other prevalent conditions, such as malaria and nutritional deficiencies, leading to frequent misdiagnosis.^[1,19-23]^

### Diagnosis of thalassemia

Diagnosing thalassemia involves a combination of clinical evaluation, laboratory tests, and genetic screening. The process is essential for confirming the presence of the disease, determining its type, and distinguishing it from other causes of anemia. Early and accurate diagnosis is critical to ensure appropriate management, prevent complications, and offer genetic counseling to affected families. In Sub-Saharan Africa, where thalassemia remains underdiagnosed due to limited resources, raising awareness about diagnostic methods is crucial for improving health outcomes^[1,19]^.

1. Clinical evaluation: The diagnosis of thalassemia often begins with a thorough clinical evaluation. Symptoms typically include fatigue, pallor, growth retardation, jaundice, and an enlarged spleen or liver (splenomegaly or hepatomegaly). In severe cases, especially in beta-thalassemia major, symptoms may present early in childhood, such as poor feeding, delayed physical development, and pale skin. The family history of anemia or genetic conditions may also provide important clues, as thalassemia is inherited in an autosomal recessive pattern. However, clinical symptoms alone are not sufficient for a definitive diagnosis, as they overlap with other causes of anemia, such as iron deficiency anemia or other hemoglobinopathies^[20]^.
2. Blood tests: Once thalassemia is suspected based on clinical signs, laboratory tests are essential to confirm the diagnosis. The first-line diagnostic test is a **complete blood count (CBC)**, which measures the number and types of blood cells. In thalassemia, the CBC often shows microcytic (small) and hypochromic (pale) red blood cells, which are characteristic of thalassemia and differentiate it from iron deficiency anemia. The **mean corpuscular volume** and **mean corpuscular hemoglobin** are typically low in thalassemia, indicating the presence of smaller and paler red blood cells. Additionally, a **reticulocyte count** may be elevated, reflecting the bone marrow’s attempt to produce more red blood cells to compensate for the anemia^[21]^.
3. Hemoglobin electrophoresis: The most important diagnostic test for confirming thalassemia is **hemoglobin electrophoresis**. This test separates the different types of hemoglobin present in the blood based on their electrical charge and migration through a gel. In patients with thalassemia, abnormal hemoglobin patterns can be identified. For example, in beta-thalassemia, there may be an increased amount of fetal hemoglobin (HbF) and an absent or reduced amount of adult hemoglobin (HbA). In alpha-thalassemia, the hemoglobin electrophoresis test may show a normal pattern, as the disease does not significantly alter the structure of hemoglobin, but other diagnostic tests are necessary to confirm the diagnosis^[22]^.
4. Iron studies: **Iron studies** are often conducted to rule out iron deficiency anemia, which shares similar symptoms with thalassemia. These tests typically include serum iron, ferritin levels, total iron-binding capacity, and transferrin saturation. In iron deficiency anemia, ferritin levels are low, while in thalassemia, iron studies are usually normal or may show iron overload, particularly in individuals who have received frequent blood transfusions. This helps differentiate between the two conditions and confirms that the anemia is not caused by iron deficiency^[23]^.
5. Genetic testing: **Genetic testing** plays a crucial role in diagnosing thalassemia, particularly in confirming the presence of mutations in the **alpha-globin** or **beta-globin** genes. For beta-thalassemia, DNA analysis can detect mutations that cause the disease, such as point mutations, deletions, or insertions. Genetic testing is especially important for identifying carriers of the thalassemia gene (thalassemia trait), who may have no symptoms but can pass the gene to their offspring. In alpha-thalassemia, genetic testing is used to detect deletions in the alpha-globin gene, which are common in many African populations. While genetic testing is highly accurate, it may not always be readily available in low-resource settings, such as parts of Sub-Saharan Africa, due to the high cost and the need for specialized equipment^[24]^.
6. Prenatal diagnosis: Prenatal diagnosis is crucial for families with a history of thalassemia or when both parents are carriers. In cases of suspected thalassemia, **chorionic villus sampling (CVS)** or **amniocentesis** can be performed to test the fetus for thalassemia mutations. These tests can detect the presence of beta-thalassemia major or alpha-thalassemia at an early stage, allowing parents to make informed decisions about the pregnancy. Early prenatal diagnosis is an essential tool for preventing the birth of children with severe forms of the disease and for providing genetic counseling to at-risk families^[25]^.
7. Newborn screening: Newborn screening for thalassemia is not widely implemented in Sub-Saharan Africa, although it could significantly reduce the burden of undiagnosed and untreated cases. In regions with high rates of thalassemia, early screening could identify infants with thalassemia major who require early intervention, such as blood transfusions, and prevent complications such as organ damage from iron overload. The implementation of newborn screening programs, alongside education for parents and health care providers, could dramatically improve outcomes for affected children^[26]^.
8. Other diagnostic methods: In some cases, advanced imaging techniques such as **magnetic resonance imaging (MRI)** may be used to assess iron overload in thalassemia patients who have received frequent blood transfusions. This helps in managing iron chelation therapy, which is used to remove excess iron from the body. Additionally, bone marrow aspiration and biopsy may be performed in rare cases to assess the marrow’s response to anemia, although this is not commonly used for routine diagnosis^[27]^.

### Management of thalassemia

The management of thalassemia requires a comprehensive approach aimed at alleviating the symptoms, preventing complications, and improving the quality of life for affected individuals. The management strategies vary based on the type and severity of thalassemia, as well as the individual’s age, health status, and access to health care resources. In Sub-Saharan Africa, where thalassemia remains underdiagnosed and health care resources are limited, managing the disease presents significant challenges. However, several treatment modalities are available, ranging from blood transfusions to stem cell transplantation, which can help improve outcomes and extend life expectancy for those with severe forms of thalassemia^[28]^.

1. Blood transfusions: Regular blood transfusions are the cornerstone of treatment for individuals with **beta-thalassemia major**, the most severe form of the disease. Blood transfusions help to alleviate the symptoms of anemia by providing healthy red blood cells, thereby preventing complications such as fatigue, poor growth, and organ dysfunction. Transfusion therapy needs to be performed regularly, typically every 2–4 weeks, depending on the patient’s individual needs. Transfusions improve the overall quality of life and prolong survival by maintaining adequate hemoglobin levels. However, repeated transfusions lead to iron overload, which can damage vital organs such as the heart, liver, and endocrine glands^[29]^.
2. Iron chelation therapy: One of the major complications of long-term blood transfusions is **iron overload**, which occurs as excess iron from the transfused blood accumulates in the body. This excess iron can damage organs, leading to severe complications such as liver cirrhosis, heart failure, and diabetes. To prevent iron overload, individuals undergoing regular transfusions must receive **iron chelation therapy**. Chelating agents, such as **deferoxamine, deferasirox,** and **deferiprone**, bind to excess iron and help the body excrete it through urine or feces. The choice of chelation therapy depends on factors such as the patient’s age, the severity of iron overload, and available treatment options. Regular monitoring of iron levels is essential to guide chelation therapy and prevent organ damage^[30]^.
3. Bone marrow/stem cell transplantation: HSCT, also known as bone marrow transplantation, offers a potential curative treatment for individuals with thalassemia, particularly for children with beta-thalassemia major. HSCT involves replacing the patient’s defective bone marrow with healthy donor stem cells, which can produce normal red blood cells. This treatment offers the possibility of a permanent cure, but it carries significant risks, including graft-versus-host disease, infections, and organ toxicity. Finding a suitable donor, often a sibling, is essential for successful transplantation. Although this treatment has shown promise in improving survival and quality of life, its availability in Sub-Saharan Africa is limited due to the high cost, need for specialized health care facilities, and lack of suitable donor registries^[31]^.
4. Gene therapy: Gene therapy is an emerging treatment option for thalassemia that aims to correct the underlying genetic defect. By introducing a healthy copy of the defective gene into the patient’s bone marrow cells, gene therapy can potentially restore normal hemoglobin production. Although gene therapy holds great promise as a potential cure for thalassemia, it is still in the experimental stage and is not widely available, particularly in resource-limited settings like Sub-Saharan Africa. Researchers are working on improving the efficiency, safety, and accessibility of gene therapy to make it a viable treatment option for thalassemia patients in the future^[32]^.
5. Splenectomy: **Splenectomy**, or the surgical removal of the spleen, may be necessary for patients with thalassemia who develop **splenomegaly** (enlargement of the spleen) due to frequent blood transfusions or excessive destruction of red blood cells. The spleen is responsible for filtering out abnormal red blood cells, and in thalassemia, it becomes enlarged and overactive. Splenectomy may help alleviate some symptoms, such as pain and reduced blood cell destruction. However, it also increases the risk of infections, and patients who undergo splenectomy require lifelong prophylactic antibiotics and immunizations to prevent infections, particularly from encapsulated bacteria^[33]^.
6. Folic acid supplementation: **Folic acid** supplementation is an important part of thalassemia management. Folic acid is a B vitamin that is essential for the production of new red blood cells. Because thalassemia patients often have increased red blood cell turnover, they require higher levels of folic acid to support the production of healthy red blood cells. Folic acid supplementation helps to prevent further anemia and supports overall blood health. It is typically recommended for individuals with both thalassemia major and minor^[34]^.
7. Management of complications: Thalassemia can lead to various complications that require management throughout a patient’s lifetime. These complications include **endocrine dysfunctions** (such as growth hormone deficiency, hypothyroidism, and diabetes), **cardiovascular issues** (such as heart failure due to iron overload), and **bone deformities** (resulting from ineffective erythropoiesis). Regular screening for these complications is essential for early intervention. For example, growth hormone therapy may be needed for children with growth retardation, while thyroid hormones or insulin may be required to manage hypothyroidism or diabetes. In severe cases, cardiac medications and devices may be necessary to manage heart failure related to iron overload^[35]^.
8. Psychosocial support: In addition to medical treatment, thalassemia patients, particularly those with severe forms, require comprehensive psychosocial support. Living with a chronic condition can be emotionally and psychologically challenging, particularly for children and their families. Psychosocial support services, including counseling, social work, and peer support groups, can help families cope with the emotional burden of managing thalassemia. It is also essential to address the financial strain that treatment, such as frequent blood transfusions and iron chelation, can place on families in low-resource settings. Community-based interventions and support programs can help provide a better quality of life for patients and their families^[36]^.
9. Preventive care and health education: Preventive care is an important aspect of managing thalassemia, particularly in Sub-Saharan Africa, where early diagnosis and treatment are not always accessible. Health education programs focused on **genetic counseling, screening**, and **prevention** of thalassemia can help reduce the incidence of severe forms of the disease. Public health initiatives aimed at raising awareness about thalassemia, as well as promoting the benefits of prenatal screening and genetic counseling, can significantly reduce the burden of the disease. Additionally, encouraging safe blood donation practices and ensuring access to quality blood transfusions can help manage patients’ health and reduce complications^[37]^.
10. **Access to Treatment in Sub-Saharan Africa**: Access to appropriate thalassemia treatment in Sub-Saharan Africa is limited by several factors, including **resource constraints, poor health care infrastructure**, and **lack of trained health care professionals**. Blood transfusions, iron chelation therapy, and advanced treatments such as stem cell transplantation are often not readily available in many parts of the region. To improve management outcomes, it is essential to invest in health care infrastructure, train health care providers in the diagnosis and management of thalassemia, and ensure the availability of essential medications. International partnerships, increased funding for thalassemia care, and improved access to affordable treatments will play a key role in enhancing the management of thalassemia in Sub-Saharan Africa^[37]^.

### Challenges in thalassemia management in Sub-Saharan Africa

Managing thalassemia in Sub-Saharan Africa presents unique challenges due to the region’s limited health care infrastructure, financial constraints, and high rates of undiagnosed or misdiagnosed cases. While thalassemia is a significant public health concern, especially among children, its management remains suboptimal due to several factors. The lack of awareness, access to diagnostic tools, and necessary treatments contribute to poor disease outcomes and high morbidity in affected individuals. These challenges must be addressed to improve the lives of individuals living with thalassemia in the region.

1. Limited diagnostic facilities: One of the most significant challenges in managing thalassemia in Sub-Saharan Africa is the lack of accessible and affordable diagnostic facilities. Diagnosing thalassemia often requires specialized blood tests, such as hemoglobin electrophoresis, DNA analysis, and family genetic screening, which may not be available in many parts of the region. Early diagnosis is crucial for initiating appropriate treatment, such as blood transfusions and iron chelation, to prevent complications. However, the absence of these diagnostic services, especially in rural or remote areas, results in delayed diagnosis and poor disease management. Many patients are often diagnosed late in life, leading to irreversible complications, including organ damage and growth failure^[38]^.
2. Inadequate access to blood transfusions: Blood transfusion is a cornerstone in the management of severe thalassemia, particularly **beta-thalassemia major**. However, the availability of safe and adequate blood supplies is a significant issue in Sub-Saharan Africa. The region often faces blood shortages due to limited donor recruitment, low awareness about voluntary blood donation, and logistical challenges in storing and distributing blood. As a result, many thalassemia patients do not receive the regular transfusions they need, leading to severe anemia, poor growth, and developmental delays. The lack of blood supply infrastructure in remote areas exacerbates this issue, making it difficult for patients to adhere to the transfusion schedule essential for their survival and well-being^[39]^.
3. Iron overload management: A critical complication of regular blood transfusions is iron overload, which can lead to organ damage, particularly in the heart, liver, and endocrine system. **Iron chelation therapy**, which is essential for removing excess iron from the body, is often inaccessible or unaffordable in Sub-Saharan Africa. Iron chelation agents like **deferoxamine, deferasirox**, and **deferiprone** are not always available in public health facilities, and their high cost makes them unaffordable for many patients, particularly those in low-income settings. Without proper iron chelation, patients risk developing severe complications, including heart failure, diabetes, and liver cirrhosis, which can significantly shorten their life expectancy^[40]^.
4. Limited access to advanced treatments: Advanced treatments for thalassemia, such as **stem cell transplantation** or **gene therapy**, offer potential cures or long-term solutions. However, these treatments remain largely unavailable in Sub-Saharan Africa due to the high cost, lack of specialized health care infrastructure, and limited access to suitable donors. HSCT, while potentially life-saving, requires a compatible donor, a well-equipped transplant center, and a skilled medical team. Unfortunately, such services are often concentrated in wealthier, urban areas or in other regions, leaving most patients in Sub-Saharan Africa without access to curative treatments. Gene therapy, although promising, is still in the research and development phase and remains far from being accessible in resource-limited settings^[41]^.
5. Financial constraints: The financial burden of managing thalassemia is a significant challenge in Sub-Saharan Africa. The costs associated with blood transfusions, iron chelation therapy, and other supportive treatments can be overwhelming, particularly for families living in poverty. In many cases, the cost of ongoing medical care is beyond the reach of most affected individuals, leading to treatment non-compliance and worsening health outcomes. Moreover, health care systems in the region often lack the resources to subsidize the cost of these essential treatments, resulting in significant barriers to access. Financial constraints also impact health care facilities, limiting their ability to invest in diagnostic tools, medications, and trained health care professionals required to manage thalassemia effectively^[42]^.
6. Lack of skilled health care providers: Managing thalassemia requires a multidisciplinary approach involving hematologists, pediatricians, endocrinologists, and other specialists. However, Sub-Saharan Africa faces a shortage of health care professionals with expertise in thalassemia management. Many health care providers are not adequately trained in recognizing or treating thalassemia, and there is a general lack of specialized hematology services. This knowledge gap leads to misdiagnosis or late diagnosis, which exacerbates the severity of the disease. Training programs for health care providers in thalassemia diagnosis and treatment are scarce, and the region’s health care workforce is often overwhelmed by a broad range of medical conditions, limiting attention to rare and complex diseases like thalassemia^[43]^.
7. Cultural and social barriers: In some parts of Sub-Saharan Africa, cultural and social barriers further complicate the management of thalassemia. In many cases, thalassemia is perceived as a genetic or familial disorder, leading to stigmatization and discrimination against affected individuals and their families. The lack of awareness about thalassemia, particularly in rural communities, can result in misconceptions about the disease, preventing families from seeking medical care. Additionally, some families may not fully understand the importance of regular blood transfusions or iron chelation therapy, leading to poor adherence to treatment protocols. Public health campaigns and educational programs are needed to raise awareness and reduce stigma, improving care-seeking behaviors and treatment compliance^[44]^.
8. Delayed or inadequate preventive measures: Preventive measures, such as **genetic counseling** and **screening programs**, are critical in reducing the incidence of severe thalassemia in Sub-Saharan Africa. However, these measures are often not implemented or are available only in urban centers, leaving rural populations with limited access to preventive care. Many people with **thalassemia minor** may unknowingly pass on the disease to their children, as genetic screening is not routinely offered. Increased efforts to integrate genetic counseling into public health programs, particularly for high-risk communities, can help reduce the burden of thalassemia and prevent the birth of children with severe forms of the disease^[45]^.
9. Lack of national health policies: The absence of national health policies specifically addressing thalassemia is another barrier to effective disease management in Sub-Saharan Africa. Many countries in the region do not have comprehensive national programs for thalassemia care, which results in fragmented and inconsistent treatment across different regions. Without coordinated efforts from governments, health care systems, and international organizations, patients in low-resource areas continue to face significant challenges in accessing quality care. National health policies that prioritize thalassemia management, integrate it into broader health care frameworks, and promote public–private partnerships could help improve outcomes for patients^[44]^.
10. Public awareness and advocacy: Public awareness and advocacy for thalassemia are limited in Sub-Saharan Africa, leading to insufficient government funding, community support, and media attention. Advocacy efforts can help highlight the challenges faced by individuals with thalassemia, create public awareness campaigns, and mobilize resources for better care and treatment. Non-governmental organizations, patient support groups, and global health initiatives can play an essential role in raising awareness and pushing for policy changes that address the needs of individuals with thalassemia in the region^[45]^.

### Innovations in thalassemia management

Innovations in thalassemia management are crucial in improving patient outcomes and addressing the unique challenges faced by individuals living with this genetic disorder, particularly in resource-limited settings like Sub-Saharan Africa. Advances in diagnostics, treatment strategies, and supportive care are transforming the way thalassemia is managed, offering new hope for affected individuals. These innovations focus on improving early diagnosis, enhancing the effectiveness of treatment regimens, and exploring potential curative therapies. This section highlights key innovations in the management of thalassemia, from diagnostic techniques to novel therapeutic approaches.

1. Advanced diagnostic techniques: One of the most significant innovations in thalassemia management is the improvement in diagnostic methods. Traditional methods, such as hemoglobin electrophoresis and CBC, are now complemented by more advanced techniques such as **high-performance liquid chromatography (HPLC), DNA sequencing**, and **PCR-based assays**. These technologies allow for early detection and accurate identification of the various forms of thalassemia, even in cases of minor or silent carrier status. Early diagnosis enables health care providers to initiate appropriate interventions, such as blood transfusions and iron chelation therapy, before severe complications develop. In Sub-Saharan Africa, the widespread use of **point-of-care diagnostic devices**, which offer quick and affordable testing, could play a key role in addressing the diagnostic gap and improving outcomes in low-resource settings^[46,47]^.
2. Gene therapy: Gene therapy represents a transformative approach to the treatment of thalassemia and holds the potential for a long-term cure. Recent advancements in **gene-editing technologies**, particularly **CRISPR-Cas9**, have opened up new possibilities for correcting the genetic mutations that cause thalassemia. By directly editing the gene responsible for the production of abnormal hemoglobin, gene therapy could potentially eliminate the need for lifelong blood transfusions and iron chelation therapy. Clinical trials using gene therapy for thalassemia have shown promising results, with some patients experiencing near-normal hemoglobin levels and a reduced dependence on transfusions. Although gene therapy is still in its experimental stages, it holds significant promise for future management, especially in high-income countries where such treatments are more accessible^[48,49]^.
3. Stem cell transplantation: **HSCT**, or bone marrow transplantation, is one of the most established curative treatments for thalassemia. Over the years, advancements in stem cell transplantation techniques, including improved matching of donors and better post-transplant care, have increased the success rates of this procedure. HSCT can provide a permanent solution by replacing the patient’s defective bone marrow with healthy, donor-derived stem cells that can produce normal red blood cells. Although stem cell transplantation remains a complex and high-risk procedure, recent innovations in reducing transplant-related complications and improving patient outcomes are enhancing its feasibility. In some African countries, collaboration with international transplant centers has enabled access to HSCT for a select group of patients, offering them a potential cure. However, the high cost, need for matched donors, and lack of specialized facilities limit the widespread use of this treatment in Sub-Saharan Africa^[50,51]^.
4. Iron chelation therapy: Iron overload due to frequent blood transfusions is a major concern for individuals with thalassemia. The development of oral iron chelators has been a significant advancement in managing this complication. Traditional iron chelation therapy requires intravenous infusion, making it inconvenient and difficult for many patients to adhere to the treatment schedule. Innovations such as **deferasirox**, an oral iron chelator, have greatly improved the quality of life for patients by offering an easier and more effective way to manage iron overload. Other newer iron chelators, including **deferiprone**, have shown promise in reducing iron levels while minimizing side effects. These innovations have made it easier for patients, especially in low- and middle-income countries, to manage the side effects of transfusions and improve long-term survival^[52]^.
5. Blood substitution and artificial blood: Although not yet a standard practice in thalassemia management, the development of **artificial blood** and **blood substitutes** offers a potential solution to the challenges of blood transfusions. Artificial blood products, such as hemoglobin-based oxygen carriers and perfluorocarbon emulsions, are designed to mimic the oxygen-carrying capacity of red blood cells. These innovations could help address the chronic shortage of blood donations, particularly in regions with limited access to safe blood products. While these blood substitutes are still in the research phase and have not yet been widely adopted, ongoing studies suggest that they may eventually provide an alternative or supplement to traditional blood transfusions, improving the quality of care for patients with thalassemia^[53,54]^.
6. Non-invasive monitoring of iron overload: Non-invasive techniques for monitoring iron overload have emerged as a promising innovation in thalassemia management. Traditional methods, such as liver biopsy, are invasive and carry significant risks. However, newer imaging techniques, such as **MRI**, have been developed to measure iron levels in the liver, heart, and other organs without the need for invasive procedures. MRI-based T2 imaging* is now widely used to assess myocardial iron burden, which is a critical factor in predicting cardiac complications in thalassemia patients. These non-invasive methods offer a safer, more accessible alternative for monitoring iron overload, allowing for earlier intervention and more personalized treatment plans^[55]^.
7. Patient-centered care models: A growing recognition of the importance of patient-centered care has led to innovations in how thalassemia is managed. Comprehensive care models that involve multidisciplinary teams, including hematologists, cardiologists, endocrinologists, and psychologists, are becoming increasingly common. These teams work together to address the diverse needs of thalassemia patients, including managing complications such as growth delays, endocrine dysfunction, and psychosocial issues. In addition, telemedicine and digital health technologies are being utilized to provide remote consultations and follow-up care, improving access to care for patients in remote or underserved areas. These innovations in care delivery ensure that thalassemia patients receive holistic, individualized treatment plans that can improve their overall quality of life^[56]^.
8. Patient and family education and support programs: Innovations in thalassemia management are also focused on improving patient and family education and providing psychosocial support. Support programs and educational campaigns are helping raise awareness about thalassemia, its complications, and the importance of early diagnosis and treatment. Patient advocacy groups and non-governmental organizations (NGOs) are playing an essential role in supporting families affected by thalassemia by providing financial assistance, emotional support, and educational resources. These programs help patients and their families navigate the complexities of thalassemia management, reduce stigma, and improve adherence to treatment protocols.^[57-59]^
9. Prenatal and genetic screening: Early detection of thalassemia through **genetic screening** and **prenatal diagnostics** has been a major innovation in preventing severe cases of the disease. Advances in prenatal testing, such as **chorionic villus sampling (CVS)** and **amniocentesis**, allow for early detection of thalassemia mutations in unborn children, providing parents with the option of informed decision-making regarding pregnancy management. In addition, carrier screening programs are increasingly being implemented in high-risk populations, which can help identify individuals with thalassemia trait before they have children. These innovations are crucial for reducing the burden of severe thalassemia in the population and promoting better health outcomes^[60]^.
10. Global collaborations and research initiatives: Finally, international collaborations and research initiatives are accelerating progress in thalassemia management. Organizations such as the **World Health Organization (WHO)** and the **Thalassemia International Federation (TIF)** are working to improve access to care and research funding in low- and middle-income countries. Collaborative research efforts are leading to the development of new therapies, improving access to treatments, and facilitating the exchange of knowledge and resources across borders. Global initiatives are essential for addressing the inequities in thalassemia care and ensuring that advancements in treatment benefit patients worldwide^[61]^.

### Prevention strategies for thalassemia

Thalassemia is a genetic blood disorder that significantly affects the quality of life for those affected. Since the condition is inherited in an autosomal recessive manner, prevention strategies primarily focus on identifying carriers of the disease, reducing the incidence of thalassemia in affected populations, and promoting early diagnosis. Effective prevention involves a combination of public health policies, education, and medical interventions aimed at reducing the number of children born with thalassemia major and reducing the burden on affected families and health care systems. Here are several prevention strategies that have been implemented or are being considered to reduce the prevalence and impact of thalassemia.

1. Carrier screening programs: Carrier screening is one of the most effective prevention strategies for thalassemia. This involves testing individuals for the presence of the thalassemia trait (heterozygous carriers) who may not show symptoms of the disease but can pass the mutated gene to their offspring. Screening programs, particularly those targeting high-risk populations, can identify couples at risk of having children with thalassemia major. Screening is typically done through blood tests, including hemoglobin electrophoresis, CBC, and more advanced molecular diagnostic tests. In many countries, pilot programs have been introduced, particularly in areas with high incidences of thalassemia, such as the Mediterranean, South Asia, and parts of Sub-Saharan Africa^[62]^.
2. Genetic counseling: Once carriers are identified through screening, genetic counseling plays a crucial role in educating them about the risk of having children with thalassemia and informing them of their options. Genetic counselors provide information about inheritance patterns, the likelihood of passing on the disorder, and possible prenatal diagnostic tests, such as amniocentesis or chorionic villus sampling (CVS), to determine whether the fetus is affected. For couples identified as high-risk (both carriers of thalassemia), counseling may offer options such as preconception counseling, reproductive choices, or prenatal testing, which can help in making informed decisions about family planning^[63]^.
3. Preconception and prenatal screening: Expanding preconception screening and prenatal screening programs can significantly reduce the incidence of thalassemia. Preconception screening, especially in populations with a high carrier rate, allows couples to understand their genetic risks before conception. If both partners are carriers of thalassemia, prenatal diagnosis can confirm whether the fetus is affected. The availability of prenatal testing empowers families to make informed choices, including the possibility of pregnancy termination, in cases of severe thalassemia major. Early diagnosis also opens up the possibility of planning for the immediate management of the condition, such as blood transfusions and other supportive therapies^[64]^.
4. Public health education and awareness campaigns: Public awareness campaigns are essential in increasing the understanding of thalassemia and its prevention. In many communities, there may be limited awareness of the condition, its inheritance patterns, and the importance of genetic screening. National and local health authorities, in collaboration with NGOs, can play a pivotal role in educating the public through media campaigns, educational materials, and community engagement programs. These campaigns can target school-age children, young adults, and individuals in high-risk communities, encouraging them to seek screening and counseling. Early education on thalassemia also helps reduce the stigma associated with the disease and promotes early intervention^[65]^.
5. Integration of thalassemia prevention into National Health Policies: To ensure long-term sustainability and success in thalassemia prevention, national health policies need to integrate strategies for thalassemia control. Governments should incorporate carrier screening, genetic counseling, and education programs into their national health agendas. By providing funding for these initiatives, including subsidies for diagnostic testing and counseling services, governments can make thalassemia prevention more accessible, especially in low-resource settings. Additionally, ensuring that health care providers are trained in diagnosing and managing thalassemia and its complications is critical in achieving the goals of these programs^[66]^.
6. Promoting marriage and reproductive decision-making: In some high-prevalence areas, culturally appropriate strategies are needed to address marriage customs and reproductive decision-making. In regions where arranged marriages are common, couples may be unaware of their genetic status. Community-based initiatives that include thalassemia education and carrier screening before marriage can play an important role in preventing the birth of children with thalassemia major. Religious and community leaders can be important partners in promoting the integration of thalassemia prevention programs into community practices and encouraging informed family planning^[67]^.
7. Gene editing and preimplantation genetic diagnosis (PGD): For couples who are both carriers of thalassemia and wish to avoid the risk of passing the disorder to their offspring, innovative techniques such as **PGD** and **gene editing** offer new possibilities. PGD, which involves screening embryos created through *in vitro* fertilization (IVF) for thalassemia mutations before implantation, enables parents to select embryos that are free of the disorder. Additionally, advances in **gene-editing technologies**, such as CRISPR-Cas9, are being explored as potential methods for editing the genetic mutations responsible for thalassemia before conception or during early embryonic development. While these techniques are still in the experimental stages, they hold promise for providing families with additional reproductive options to prevent thalassemia^[68]^.
8. Improved blood donation systems: As blood transfusions are a major aspect of managing thalassemia, efforts to improve blood donation systems in regions with a high prevalence of thalassemia can indirectly contribute to better management and prevention. Encouraging voluntary blood donations and improving blood donation infrastructure is crucial to ensure that thalassemia patients have access to safe and adequate blood supplies for transfusions. Many countries have introduced donor mobilization campaigns to promote voluntary blood donation, which is particularly important in areas with high rates of thalassemia, where transfusion dependency is a significant burden^[69]^.
9. Regional and international collaboration: Regional and international collaborations are vital in the prevention and management of thalassemia. Developing partnerships with global organizations like the **WHO, TIF**, and other research institutions helps mobilize resources, enhance research on thalassemia prevention, and implement best practices across borders. Collaborative efforts can improve access to diagnostic tools, establish uniform screening protocols, and share knowledge on effective prevention strategies tailored to specific regions or populations. Countries with limited resources can benefit from knowledge exchange with countries that have successfully implemented prevention programs, leading to a broader and more effective approach to thalassemia prevention in Sub-Saharan Africa and other parts of the world^[70]^.
10. Legislative measures: In some countries, the introduction of legislation to mandate thalassemia screening at the national level can be an effective preventive measure. For example, laws that require thalassemia screening for couples before marriage or before having children can significantly reduce the number of births with the disease. Legislative measures can also ensure that health care systems are adequately equipped with the necessary tools and services, such as affordable genetic testing, counseling, and support for affected families. Legal frameworks that promote thalassemia education and prevention programs can ensure long-term and sustainable control of the disorder^[71]^.

### The role of government and policy in thalassemia care

Governments play a crucial role in shaping the health care infrastructure, funding, and policies that directly affect the diagnosis, treatment, and prevention of thalassemia. Due to the genetic nature of the disease and the need for lifelong care, a robust, well-supported policy framework is essential to ensure that individuals with thalassemia receive appropriate care and support. The role of government in thalassemia care involves several key areas, including public health initiatives, access to health care services, education and awareness programs, and financial support for affected families. Effective government action can drastically improve both the quality of care for patients and the prevention of new cases through genetic screening and educational initiatives.

1. National screening programs: One of the most significant roles of governments in thalassemia care is the implementation of national carrier screening programs. Thalassemia is a genetically inherited disease, and screening for carrier status can help reduce the incidence of thalassemia major. Governments can support these efforts by integrating genetic screening into public health policies, particularly in areas where thalassemia is endemic. Nationwide screening initiatives, which include blood tests and genetic counseling, enable early identification of carriers, providing couples with the necessary information to make informed decisions about family planning. Policies that mandate or incentivize screening before marriage, or during the early stages of pregnancy, can reduce the number of children born with severe forms of thalassemia^[72]^.
2. Access to treatment and medications: The management of thalassemia requires lifelong blood transfusions, iron chelation therapy, and sometimes bone marrow transplants. Governments are responsible for ensuring that these treatments are accessible, affordable, and of high quality. National health policies should allocate sufficient resources to provide these essential treatments to all patients, regardless of their socioeconomic status. For example, governments can subsidize the cost of blood transfusions, iron chelation drugs, and other medical interventions needed for thalassemia care. Ensuring that thalassemia patients have access to these therapies can help reduce the burden of the disease and improve patients’ quality of life. Additionally, governments should invest in specialized medical centers and training programs for health care professionals to manage thalassemia effectively^[73]^.
3. Education and awareness campaigns: Governments are instrumental in increasing public awareness of thalassemia through nationwide education campaigns. These programs can help inform the general population about the risks of thalassemia, the importance of genetic screening, and the need for regular monitoring and treatment. Public awareness campaigns can also reduce the stigma surrounding the disease, encouraging more people to seek care and get screened for carrier status. Educational initiatives targeting schools, community groups, and health care providers can promote a deeper understanding of thalassemia, especially in regions where the disease is prevalent. By working with schools, local leaders, and community health organizations, governments can facilitate widespread education and prevent misperceptions about the disease^[74]^.
4. Policy integration into primary health care systems: Governments can integrate thalassemia care into national primary health care systems, ensuring that individuals have access to early diagnosis, ongoing monitoring, and appropriate treatment. Including thalassemia in the essential health services package can help make care more accessible, especially in rural or under-resourced areas. Local health centers, supported by government policy, can provide basic care, screenings, and referrals to specialized centers. Primary health care providers should be trained to recognize early signs of thalassemia and refer patients for further diagnostic testing, ensuring that individuals receive timely care. By integrating thalassemia management into routine health care services, governments can increase the reach and effectiveness of treatment across the population^[75]^.
5. Research and data collection: Governments have a responsibility to fund research on thalassemia, including studies on genetic mutations, treatment advancements, and cost-effective interventions. Research initiatives that aim to better understand thalassemia’s epidemiology and pathophysiology can guide public health policies and clinical practices. Governments can also support the establishment of national thalassemia registries to track patient outcomes, treatment efficacy, and disease progression. Data collection allows for more accurate predictions of the disease burden, informing resource allocation and the development of more effective public health strategies. Additionally, collaboration with international organizations and research institutions can enhance the quality of research and facilitate the sharing of best practices in thalassemia care.
6. Financial support and health insurance: The high cost of thalassemia treatment, particularly blood transfusions, iron chelation therapy, and bone marrow transplants, can place a significant financial strain on families affected by the disease. Government policy should include provisions for financial support to reduce the economic burden of thalassemia. This support could come in the form of subsidized medications, free or low-cost blood transfusions, or health insurance coverage for patients. Governments can also provide direct financial assistance to families who require ongoing care or support for their children with thalassemia. Financial assistance programs help ensure that thalassemia patients can access life-saving treatments without facing financial hardship, thus improving overall disease outcomes^[71]^.
7. Collaboration with international organizations: Governments can collaborate with international health organizations such as the **WHO, TIF**, and other NGOs to improve thalassemia care. These collaborations can provide technical support, funding for health care initiatives, and help implement best practices in disease management. International partnerships can also facilitate the sharing of knowledge and resources, helping to improve national health systems and enhance the quality of care provided to thalassemia patients. By working together with global institutions, governments can access expertise and resources that might not be readily available within their borders^[72]^.
8. Legal frameworks for reproductive health: Governments can enact legal frameworks that support reproductive health choices for individuals at risk of passing on thalassemia to their children. Legislation that mandates genetic screening before marriage or childbirth can reduce the incidence of thalassemia major by identifying carriers early on. Additionally, laws that support reproductive health services, such as access to prenatal testing, PGD, and counseling, can enable families to make informed decisions about having children. By integrating thalassemia prevention into broader reproductive health policies, governments can empower individuals to make choices that minimize the risk of passing on genetic disorders^[73,74]^.
9. Public–private partnerships: Governments can also engage in public–private partnerships to improve the delivery of thalassemia care. Collaborations with pharmaceutical companies, private hospitals, and research institutions can help secure resources for thalassemia treatment, including the provision of medications, medical devices, and blood transfusion supplies. Such partnerships can help fill gaps in public health care systems, especially in low-resource settings, and ensure that thalassemia care is available across different economic strata. Furthermore, the private sector can assist in the development and distribution of new therapies and innovations in thalassemia care, contributing to the ongoing advancement of treatment options^[75]^.

### Future directions in thalassemia management in Sub-Saharan Africa

The future of thalassemia management in Sub-Saharan Africa lies in the integration of innovative strategies, cutting-edge research, and comprehensive public health initiatives. While the current landscape presents numerous challenges, significant advancements in medical research and health care infrastructure offer promising solutions that could transform the care of individuals living with thalassemia. Several key directions are poised to reshape the future of thalassemia care in the region, focusing on early detection, personalized treatment options, genetic counseling, and policy reforms^[50,76]^.

1. Advancements in genetic screening and diagnosis: The future of thalassemia management in Sub-Saharan Africa hinges on the widespread implementation of advanced genetic screening programs. As the understanding of the genetic mutations responsible for thalassemia deepens, more accessible and affordable genetic tests will be developed. These advancements can allow for earlier detection of carriers and provide the opportunity for genetic counseling before and during pregnancy. In addition, the development of point-of-care diagnostic tools could significantly reduce the time and costs associated with diagnosing thalassemia. Integrating these technologies into public health programs would allow for comprehensive screening at the community level, particularly in rural or underserved regions, where the prevalence of thalassemia is higher^[77]^.
2. Innovative treatment options: Traditional treatment methods for thalassemia, such as regular blood transfusions and iron chelation therapy, remain resource-intensive and require lifelong management. However, the future of thalassemia care will likely see the development of more effective and less invasive treatments. One promising approach is gene therapy, which aims to correct the genetic defect at the root of thalassemia. Clinical trials and research on gene editing techniques, such as CRISPR, have shown potential in providing permanent solutions to genetic disorders like thalassemia. Although still in the experimental stages, these therapies could offer a long-term cure for individuals with thalassemia, reducing or eliminating the need for regular blood transfusions and improving the quality of life for patients^[78]^.
3. Bone marrow and stem cell transplantation: Bone marrow and stem cell transplantation remain one of the most effective treatment options for thalassemia, particularly for patients who are unable to manage the disease through blood transfusions. The future of thalassemia management will likely see more widespread access to bone marrow and stem cell transplantation, facilitated by improvements in health care infrastructure and better donor-matching techniques. Research into alternative sources of stem cells, such as umbilical cord blood or induced pluripotent stem cells (iPSCs), holds great promise for increasing the availability of transplants and overcoming challenges related to donor compatibility. Additionally, the development of less toxic conditioning regimens for stem cell transplants could improve outcomes and reduce complications^[47,79]^.
4. Prevention through public health initiatives: Prevention remains the most effective way to reduce the burden of thalassemia in Sub-Saharan Africa. Government-supported public health initiatives focused on education, genetic counseling, and carrier screening are essential for reducing the incidence of new cases. In the future, prevention strategies may include nationwide campaigns that educate the public about the risks of thalassemia and promote genetic screening before marriage or childbirth. Policy reforms that support the integration of thalassemia prevention into national reproductive health programs could play a pivotal role in decreasing the prevalence of the disease. Furthermore, innovative strategies such as genetic counseling services at the community level could empower families to make informed decisions about their reproductive health^[80]^.
5. Improved access to treatment: Expanding access to treatment remains a critical issue in Sub-Saharan Africa, where limited health care resources can often hinder the availability of necessary therapies. In the future, improving access to life-saving treatments, such as blood transfusions and iron chelation therapy, will require substantial investment in health care infrastructure, both in urban and rural areas. Partnerships between governments, NGOs, and international health agencies can help overcome barriers related to drug availability and affordability. Moreover, the development of low-cost alternatives to existing treatments could make them more accessible to a broader population. For example, efforts to produce locally manufactured iron chelation medications could reduce the financial burden on families and health care systems^[81]^.
6. Regional and international collaboration: Thalassemia is a global health challenge that requires coordinated efforts at the regional and international levels. In the future, countries in Sub-Saharan Africa could benefit from increased collaboration with international organizations, academic institutions, and thalassemia advocacy groups. These collaborations can facilitate the sharing of knowledge, resources, and expertise, ensuring that patients in the region have access to the latest treatment options and research findings. Regional partnerships could also foster the establishment of specialized thalassemia centers of excellence, providing patients with access to comprehensive care and treatment options that are currently unavailable in many parts of the region^[82]^.
7. Capacity building and training for health care professionals: To ensure the effective management of thalassemia in the long term, there is a need to strengthen the capacity of health care professionals across Sub-Saharan Africa. Future efforts should focus on training doctors, nurses, and laboratory technicians in the latest diagnostic and therapeutic techniques for thalassemia. Specialized training in hematology and genetic counseling could empower local health care providers to identify and manage thalassemia more effectively. Furthermore, building local expertise in bone marrow transplantation, gene therapy, and stem cell research could reduce the reliance on foreign expertise and make advanced treatments more accessible to African populations^[83]^.
8. Improved blood supply and transfusion safety: Ensuring a stable and safe blood supply is essential for the management of thalassemia, which often requires frequent blood transfusions. In the future, Sub-Saharan Africa could benefit from advancements in blood banking systems, including improved donor recruitment, screening processes, and transfusion practices. Additionally, the establishment of national blood transfusion services and blood banks could help mitigate the shortage of blood supplies, which currently limits treatment options for thalassemia patients. The implementation of transfusion safety protocols, such as rigorous screening for infectious diseases, could improve the safety of blood products and reduce the risk of complications associated with transfusions^[84]^.
9. Telemedicine and digital health solutions: The rise of digital health technologies presents an opportunity to expand access to thalassemia care, particularly in remote or underserved areas. In the future, telemedicine could become a key tool for providing remote consultations, monitoring treatment adherence, and offering counseling services to patients and families. Digital platforms could also enable the creation of thalassemia registries, allowing for better tracking of patient outcomes and improving clinical decision-making. Additionally, mobile health applications could provide patients with real-time access to treatment guidelines, medication reminders, and educational resources.
10. Long-term follow-up and support for patients: Long-term follow-up care is crucial for individuals with thalassemia, as the disease requires ongoing management throughout the patient’s life. Future efforts should focus on the development of comprehensive care models that include regular monitoring, psychological support, and social services. These services could help address the emotional, financial, and social challenges faced by thalassemia patients and their families. Furthermore, the establishment of patient support groups and advocacy networks could provide individuals with a platform for sharing experiences, accessing resources, and advocating for better care policies.

## Conclusion

Thalassemia remains a significant health challenge in Sub-Saharan Africa, with a high burden of disease due to genetic predisposition, limited health care infrastructure, and inadequate access to diagnostic and treatment services. Despite these challenges, there is growing hope as advancements in diagnostic technologies, treatment modalities, and public health strategies continue to emerge. The region is witnessing gradual improvements in thalassemia management, with innovations such as genetic screening, gene therapy, and stem cell transplantation offering the potential for more effective and sustainable care. The key to addressing the thalassemia burden in Sub-Saharan Africa lies in a multifaceted approach that includes early detection, comprehensive treatment strategies, and prevention through public health initiatives. Strengthening health care infrastructure, enhancing the training of health care professionals, and improving access to life-saving therapies are essential steps toward better management of thalassemia. In addition, raising awareness about the disease and promoting genetic counseling will be vital in preventing new cases through informed reproductive choices.

## Data Availability

Not applicable as this is a review.

## Research registration unique identifying number (UIN)

Not applicable as this is a review.
